# Influence of ABCB1 gene polymorphism on concentration to dose ratio and adverse effects of tacrolimus in Pakistani liver transplant recipients

**DOI:** 10.12669/pjms.37.3.3898

**Published:** 2021

**Authors:** Fahad Azam, Moosa Khan, Tanwir Khaliq, Abu Bakar Hafeez Bhatti

**Affiliations:** 1 Dr. Fahad Azam, M.Phil, Associate Professor, Pharmacology & Therapeutics, Shifa Tameer-e-Millat University, Islamabad; 2 Dr. Moosa Khan, PhD, Professor, Pharmacology and Therapeutics, Shaheed Zulfiqar Ali Bhutto Medical University, Islamabad; 3 Dr. Tanwir Khaliq, FCPS, Professor, Department of Surgery, Shaheed Zulfiqar Ali Bhutto Medical University, Islamabad; 4 Dr. Abu Bakar Hafeez Bhatti, FCPS, FRCS Consultant, Liver Transplant, Shifa International Hospital, Islamabad

**Keywords:** Adverse effects, Immunosuppression, Liver transplant, Tacrolimus

## Abstract

**Objective:**

To evaluate the possible association of ABCB1 single nucleotide polymorphism (SNPs) of the ABCB1 gene with tacrolimus dosages, concentration-to-dose ratios (CDR) and adverse effects in Pakistani liver transplant recipients.

**Methods:**

This observational study was conducted at Shifa International Hospital, Shifa Tameer-e-Millat University, Islamabad and Basic Medical Sciences Institute, Karachi from September 2016 to July 2020. Eighty-one liver transplant recipients were included. Demographics, clinical data, tacrolimus trough levels and doses were monitored. Electrochemiluminescence immunoassay (ECLIA) was used to measure tacrolimus trough levels. Transplant recipients were genotyped for three ABCB1 SNPs (rs1045642, rs2032582 and rs1128503). Acute cellular rejection (ACR), sepsis and other adverse events were monitored.

**Results:**

ABCB1 rs1045642 CC genotype showed lower tacrolimus CDR as compared to CT and TT genotype in the first week of the post-transplantation period (p=0.02). There was a significant association of polymorphisms in rs1045642, rs2032582 and rs1128503 with psychosis, sepsis and ACR respectively.

**Conclusion:**

Identification of ABCB1 rs1045642 polymorphism may shorten the time to achieve optimum levels of tacrolimus during dose titration. ABCB1 polymorphism rs1045642, rs2032582 and rs1128503 may predict adverse effects in liver transplant recipients receiving tacrolimus.

## INTRODUCTION

Liver transplant (LT) is a curative procedure for end-stage liver disease and is followed by immune suppression therapy in transplant recipients.^1^ The use of tacrolimus for post-liver transplant immune suppression was first reported in the year 1989; since then, tacrolimus has become a popular post solid organ transplantation immunosuppressant agent and is also used in the treatment of autoimmune disorders by using oral, sublingual or intravenous route. It acts by blocking calcium-dependent phosphatase enzyme calcineurin, thereby suppressing signal 2 which is required for T cell activation.^2^

Tacrolimus is preferred in LT recipients owing to high potency; however, its use is subject to narrow therapeutic index, inter-individual variability resulting in altered concentration-to-dose-ratio (CDR) and serious adverse effects such as neuro-and nephrotoxicity necessitating therapeutic drug monitoring of tacrolimus in LT recipients.^3^ Despite close monitoring and keeping blood levels within the optimal range, tacrolimus is associated with adverse effects or may result in a lack of efficacy suggesting a possible influence of genetic polymorphism on inter-individual variability in clinical response.^4^ Genetic polymorphism in cytochrome P4503A4, cytochrome P4503A5 and ABCB1 genes has been found to be associated with variable clinical response of tacrolimus in transplant recipients.^5^,^6^

An important product of the ATP-binding cassette transporter (ABCB1) gene is P-glycoprotein (P-gp) which is an efflux transporter and regulates oral absorption of many drugs and endogenous substances; ABCB1 polymorphism could therefore result in altered P-gp expression and variable clinical response to tacrolimus.^6^ The synonymous rs1045642A single-nucleotide polymorphism (SNP) of the ABCB1 gene in exon 26, 3435C>T may affect folding of the P- glycoprotein thereby changing the specificity of the substrate and has therefore been implicated for inter-individual variability in clinical response of tacrolimus.^7^ Transplant recipients homozygous for this variation would consequently have decreased expression of intestinal P-gp and this SNP has been reported widely in 40-45% Caucasians and less frequently in other ethnicities. Association of this polymorphism by linkage disequilibrium with other SNPs rs1128503 1236C>T and rs2032582 G2677T/A, has also been reported which could eventually result in altered plasma levels of tacrolimus.^8^

Administration of personalized doses of tacrolimus may reduce the time to achieve optimal levels of tacrolimus with minimum adverse effects in the clinical setting. Currently, we do not have any data regarding relevant polymorphisms especially the ABCB1 gene and its effects on trough levels of tacrolimus in the early post-transplantation period in the Pakistani population. With this background, the present study was conducted to evaluate the influence of ABCB1 gene polymorphisms on tacrolimus dosage and adverse effects in Pakistani liver transplant recipients.

## METHODS

The study was conducted at Shifa Tameer-e-Millat University, Shifa International Hospital Islamabad and Basic Medical Sciences Institute Karachi from September 2016 to July 2020 after taking approval from the Institutional Review Board of Shifa Tameer-e-Millat University (IRB#638-086-2016 dated 29-06-2016). This was an observational prospective study and eighty-one patients were enrolled after obtaining written informed consent. All liver transplantations were performed after seeking approval from the Human Organ Transplant Authority (HOTA) Pakistan and were conducted in accordance with the Helsinki Declaration. Eighty-one patients (sixty male and twenty-one female) patients were enrolled. The mean age of the patients was 47.82±10.02 Baseline characteristics, demographic data and laboratory investigations were recorded for all transplant recipients. Details of the evaluation process of the donors and recipients have been reported elsewhere.^9^,^10^

Immunosuppression was initiated 8-12 hours post-transplantation through oral or nasogastric route. Tacrolimus and steroids were the standard drugs to initiate immunosuppression; tacrolimus was initiated with 0.5 mg/day on the first-day post-transplantation. Venous blood samples of patients were collected in 5 mL EDTA plastic tubes. Electrochemiluminescence immunoassay (ECLIA) was used to measure tacrolimus trough levels and adjustments were made in the dosing of tacrolimus based on relevant clinical indices, adverse effects and CDR.^11^ Bilirubin, AST, ALT, creatinine and albumin were evaluated on daily basis. Criteria for diagnosing nephrotoxicity were serum creatinine levels ≥1.45 mg/dl; neurotoxicity manifested as seizures, tremors or psychosis; sepsis as signs of consistent fever, increased WBC count and the risk of organ dysfunction. Acute cellular rejection (ACR) was a diagnosis of exclusion and was diagnosed on abrupt derangement of liver enzymes when all probable causes of transaminitis were ruled out.

Genomic DNA was isolated using the standard proteinase K and phenol extraction method. The sequences of primers of candidate genes were downloaded from the NCBI dbSNP database (https://www.ncbi.nlm.nih.gov/snp) and were designed as follows: ABCB1 rs1045642, (forward 5′GATCTGTGAACTCTTGTTTTCA3′ and reverse 5′GAAGAGAGACTTACATTAGGC3′); ABCB1 rs2032582 (forward 5′TCAGCATTCTGAAGTCATGGAA3′ and reverse 5′TTAGAGCATAGTAAGCAGTAGGGAGT3′); ABCB1 rs1128503 (forward 5′ TCTTTGTCACTTTATCCAGC3′ and reverse 5′ TCTCACCATCCCCTCTGT3′).

PCR mixture consisted of 12μl 12.5mM PCR Master Mix (5x FIREpol^®^ Solis BioDyne), 2μl genomic DNA, 1μl forward and reverse primer each and 9μl PCR water in a final volume of 25μl. PCR was performed in a GeneAmp PCR system, Thermal Cycler BioRAD T100(USA) for all SNPs under these conditions: initial denaturation at 95ºC for five minutes followed by thirty-five cycles of denaturation at 95ºC for 30 seconds, annealing of primers at 60ºC for thirty seconds and primer extension at 72ºC for 30 seconds and final step at 72ºC for ten minutes. Reaction products were analyzed by gel electrophoresis on 2% agarose gel. The PCR products of SNPs rs1045642, rs2032582 and rs1128503 in ABCB1 were then submitted to MboI, Bseyl and Eco01091 restriction enzymes.

ABCB1 rs1045642 amplified fragment length was 244bp and cleaved by MboI into fragments of 175 and 69bp with subsequent PCR products of CC, CT and TT genotypes. ABCB1 rs2032582 with 485bp was cleaved by Bseyl into fragments of 384 and 101 with PCR products of GG, GT and TT and ABCB1 rs1128503 with fragment length of 502bp was cleaved by Eco01091 into 382 and 120 fragments with CC, CT and TT PCR products.

Quantitative variables were expressed as Mean ± Standard deviation (SD). Categorical variables were presented as values with percentages and compared by using Pearson’s χ^2^ tests. Genotype data analysis were performed by using SPSS version 23.0 (SPSS Inc., Chicago, IL, USA). A two-tailed *p*-value<0.05 was considered significant.

## RESULTS

Mean body mass index of study participants was 26.35±4.67 kg/m^2^. Sixty out of the eighty-one recipients were male and the most common aetiology was HCV. The patients’ demographic and clinical characteristics are presented in Table-I. CC genotype individuals at rs1045642 had significantly less CDR in comparison to other genotypes during week 1 *(p=*0.02*)*. A comparison of CDR of participants based on pharmacogenetic testing of ABCB1 rs1045642, rs2032582 and rs1128503 genotype are shown in Table-II.

**Table-I:** Demographic and clinical characteristics.

Variables	Recipients (N=81) (Mean±SD)
Age(years)	47.82±10.02
BMI(kg/m^2)^	26.35±4.67
MELD score	21.77±5.39
***Tacrolimus (CDR) (ng/ml/mg/day)***	
Week-1	2.39±2.02
Week-2	3.33±2.27
Week-3	1.99±1.19
Week-4	2.01±0.90
***Etiology***	***n(%)***
HCV	40(49.38)
HBV, ESLD	12(14.81)
HBV, HDV	12(14.81)
HDV	8(9.87)
Cryptogenic liver cirrhosis, HCC	2(2.46)
Other	7(8.64)
***Demographic data***	***n(%)***
Male/female	60(74.07) / 21(25.92)
***Ethnicity***	
Punjabi	30(37)
Pathan	18(22.2)
Sindhi	13(16)
Urdu speaking	11(13.6)
Others	9(11.1)

**Table-II:** Tacrolimus CDR according to recipients’ genotype.

rs numbers	Weeks	CDR of Recipients (Mean±SD) (ng/ml/mg/day) (N=81)			*p-*value

		*CC(n=18)*	*CT(n=30)*	*TT(n=33)*	
rs1045642	Week-1	1.36±0.91	3.02±2.03	2.39±2.26	0.02*
	Week-2	3.14±1.69	4.02±2.77	2.83±1.94	0.10
	Week-3	1.89±1.29	2.16±1.24	1.90±1.12	0.64
	Week-4	1.98±0.79	2.10±0.95	1.95±0.94	0.80
	Mean	2.09±0.88	2.82±1.17	2.27±1.08	0.04*

		*GG(n=9)*	*GT(n=33)*	*TT(n=39)*	

rs2032582	Week-1	2.77±2.54	2.20±1.54	2.47±2.28	0.72
	Week-2	2.40±1.64	3.33±2.15	3.56±2.49	0.39
	Week-3	1.15±0.56	2.05±1.0	2.14±1.38	0.08
	Week-4	1.70±0.75	2.0±0.91	2.10±0.94	0.50
	Mean	2.00±0.87	2.40±0.997	2.56±1.23	0.39

		*CC(n=4)*	*CT(n=37)*	*TT(n=40)*	

rs1128503	Week-1	2.05±2.44	2.39±2.33	2.43±1.71	0.94
	Week-2	2.44±0.99	3.17±1.97	3.58±2.61	0.54
	Week-3	1.10±0.41	2.04±1.26	2.04±1.15	0.32
	Week-4	1.64±0.66	2.03±0.91	2.03±0.93	0.70
	Mean	1.81±0.74	2.41±0.94	2.52±1.27	0.47

* (statistically significant)

Polymorphism in rs2032582 showed a significant association with sepsis *(p=*0.002*)*, rs1045642 with psychosis (*(p=*0.002*)* and rs1128503 polymorphism with higher incidence of ACR *(p=*0.04*)*. Details of adverse effects and ACR with reference to ABCB1 genotype polymorphism are provided in Table-III.

**Table-III:** Tacrolimus adverse effects according to recipients’ genotypes.

Genotype	Adverse Effects (n)	Genotype n (%) (N=81)			p-value

		CC (n=18)	CT (n=30)	TT (n=33)	
rs1045642	Adverse effects (+) (28)	3(3.70)	13(16.04)	12(14.81)	0.14
	Adverse effects (-) (53)	15(18.51)	17(20.98)	21(25.92)	
	Psychosis (+) (18)	1(1.23)	13(16.04)	4(4.93)	0.002*
	Psychosis (-) (63)	17(20.98)	17(20.98)	29(35.80)	
	Seizures (+) (11)	2(2.46)	6(7.40)	3(3.70)	0.46
	Seizures (-) (70)	16(19.75)	24(29.62)	30(37.0)	
	Nephrotoxicity (+) (12)	2(2.46)	4(4.93)	6(7.40)	0.79
	Nephrotoxicity (-) (69)	16(19.75)	26(32.09)	27(33.33)	
	ACR (+) (13)	4(4.93)	6(7.40)	3(3.70)	0.37
	ACR (-) (68)	14(17.28)	24(29.62)	30(37.0)	
	Sepsis (+) (20)	2(2.46)	9(11.11)	9(11.11)	0.34
	Sepsis (-) (61)	16(19.75)	21(25.92)	24(29.62)	

		*GG(n=9)*	*GT(n=33)*	*TT(n=39)*	

rs2032582	Adverse effects (+) (28)	1(1.23)	11(13.58)	16(19.75)	0.26
	Adverse effects (-) (53)	8(9.87)	22 (27.16)	23(28.39)	
	Psychosis (+) (18)	1(1.23)	8(9.87)	9(11.11)	0.86
	Psychosis (-) (63)	8(9.87)	25 (30.86)	30(37.0)	
	Seizures (+) (11)	0	5(6.17)	6(7.40)	0.67
	Seizures (-) (70)	9(11.11)	28(34.56)	33 (40.74)	
	Nephrotoxicity (+) (12)	1(1.23)	4(4.93)	7(8.64)	0.82
	Nephrotoxicity (-) (69)	8(9.87)	29(35.80)	32(39.50)	
	ACR (+) (13)	3(3.70)	5(6.17)	5(6.17)	0.32
	ACR (-) (68)	6(7.40)	28(34.56)	34(41.97)	
	Sepsis (+) (20)	2(2.46)	2(2.46)	16(19.75)	0.002*
	Sepsis (-) (61)	7(8.64)	31(38.27)	23(28.39)	

		CC(n=4)	CT(n=37)	TT(n=40)	

rs1128503	Adverse effects (+) (28)	1(1.23)	13(16.04)	14(17.28)	1.0
	Adverse effects (-) (53)	3(3.70)	24(29.62)	26(32.09)	
	Psychosis (+) (18)	1(1.23)	8(9.87)	9(11.11)	1.0
	Psychosis (-) (63)	3(3.70)	29(35.80)	31(38.27)	
	Seizures (+) (11)	1(1.23)	7(8.64)	3(3.70)	0.21
	Seizures (-) (70)	3(3.70)	30(37.03)	37(45.67)	
	Nephrotoxicity (+) (12)	0	5(6.17)	7(8.64)	0.88
	Nephrotoxicity (-) (69)	4(4.93)	32(39.50)	33(40.74)	
	ACR (+) (13)	2(2.46)	8(9.87)	3(3.70)	0.04*
	ACR (-) (68)	2(2.46)	29()	37(45.67)	
	Sepsis (+) (20)	0	7(8.64)	13(16.04)	0.22
	Sepsis (-) (61)	4(4.93)	30(37.0)	27(33.33)	

* (statistically significant).

## DISCUSSION

Genetic polymorphism associated with genes regulating absorption and metabolism of calcineurin inhibitors has an important contribution towards unpredictable response which eventually results in a longer time required to achieve titration to optimal doses.^12^ Identification of specific polymorphisms mediating high interindividual variability in plasma concentration and clinical response will facilitate dosing titration and reduce the time to reach optimal dosing with less risk of developing serious adverse effects.^13^

The present study measured and compared dose-adjusted tacrolimus concentrations in liver transplant recipients with ABCB1 gene polymorphism of ABCB1 rs1045642(C3435T), rs2032582(G2677T) and rs1128503(C1236T). To our knowledge, this is the first study reporting association of ABCB1 polymorphism with efficacy and adverse effects of tacrolimus in liver transplant recipients in Pakistani population.

The higher number of male recipients in our study is in agreement with similar studies reporting a higher number of male recipients due to an imperfect model for end-stage-liver-disease (MELD) score.^14^

In our study, the ABCB1 rs1045642 CC genotype showed lower tacrolimus concentration-to-dose ratio (CDR) in post-transplantation period week-1 (*p*=0.02). Consistent with our findings, a study reported higher tacrolimus dose requirement in recipients with CC genotype as compared to those with CT and TT genotype on first day, third day and three months follow-up post-transplantation period.^7^

Other research studies on Chinese liver-transplant recipients have found a higher dose-adjusted tacrolimus CDR at different post-transplantation periods in recipients carrying the ABCB1 T-allele as compared to recipients with homozygous CC genotype of ABCB1 C3435T.^15^ Factors responsible for variation in bioavailability of tacrolimus could be the fact that homozygous 3435CC variant of ABCB1 gene exhibits significantly lower expression of intestinal P-gp as compared to individuals with ABCB1 3435TT genotype thus affecting bioavailability of tacrolimus.^5^,^16^-^17^

According to our findings, transplant recipients with polymorphism in rs2032582 and rs1128503 had consistently higher levels of CDR throughout week-1 to week-4 although these findings were not statistically significant. Our findings are in agreement with previous studies that did not find a significant association of ABCB1 G2677T/A and C1236T polymorphism on tacrolimus pharmacokinetic parameters during the first six months in renal and liver transplant recipients. Nonetheless, another study has reported high dose tacrolimus requirements in kidney transplant recipients who were carriers of the ABCB1 2677GG wild-type genotype.^18^,^19^

High plasma levels of tacrolimus have the potential to precipitate neurotoxicity, nephrotoxicity, sepsis and gastrointestinal adverse effects in the post-transplantation period.^20^ We found a significant association of polymorphism in rs1045642 with psychosis which is consistent with our earlier findings of a higher incidence of psychosis in liver transplant recipients with higher tacrolimus levels.^21^ These findings are in agreement with previous studies which have shown a higher incidence of tacrolimus-related neurotoxicity in the presence of T allele rs1045642.^22^

Recipients with TT polymorphism in rs2032582 showed a significant association with sepsis which is in agreement with our previous findings showing a higher incidence of sepsis in recipients with higher blood tacrolimus levels.^21^ As tacrolimus is a potent immune suppressant drug therefore higher levels of this drug may increase the risk of contracting infections and sepsis.

Recipients with C>T polymorphism in rs1128503 had a higher incidence of ACR which was statistically significant. Studies in agreement with our results have shown that lower trough levels of tacrolimus are significantly associated with increased risk of ACR. 23,24 This finding is also in agreement with our earlier findings showing a higher incidence of ACR in recipients with low blood levels of tacrolimus.^21^

### Limitations of the study:

The effects of genetic polymorphism of candidate genes on long term adverse effects of tacrolimus were not observed which is one possible limitation of our study.

## CONCLUSION

The present study concludes that polymorphism in the ABCB1 rs1045642 may result in variation in tacrolimus dose requirement during dose titration and clinical efficacy. Significant association of polymorphism in rs1045642 with neurotoxicity, rs2032582 with sepsis and rs1128503 with ACR in liver transplantation recipients is found in Pakistani liver transplant recipients. Based on our results, we propose that identifying polymorphism of ABCB1 rs1045642 may shorten the time to achieve optimum levels of tacrolimus during dose titration. In future studies, the effect of polymorphism on long term adverse effects of tacrolimus should be explored.

### Authors’ Contribution:

**FA:** Conceived and designed study; collected, analyzed and interpreted data; drafted manuscript.

**MK and TK:** Designed study; interpreted data and drafted manuscript, reviewed final draft.

**ABH:** Designed study; analyzed and interpreted data; reviewed final draft.

All authors are responsible and accountable for the accuracy and integrity of the work.
